# Nephropathy in *Pparg*-null mice highlights PPARγ systemic activities in metabolism and in the immune system

**DOI:** 10.1371/journal.pone.0171474

**Published:** 2017-02-09

**Authors:** Barbara Toffoli, Federica Gilardi, Carine Winkler, Magnus Soderberg, Laura Kowalczuk, Yvan Arsenijevic, Krister Bamberg, Olivier Bonny, Béatrice Desvergne

**Affiliations:** 1 Center for Integrative Genomics, Faculty of Biology and Medicine, University of Lausanne, Lausanne, Switzerland; 2 AstraZeneca R&D, Pepparredsleden 1, Mölndal, Sweden; 3 Unit of Gene Therapy & Stem Cell Biology, University of Lausanne, Department of Ophthalmology, Fondation Asile des Aveugles, Jules-Gonin Eye Hospital, Lausanne, Switzerland; 4 Service of Nephrology, Lausanne University Hospital and Department of Pharmacology and Toxicology, University of Lausanne, Lausanne, Switzerland; University of Louisville, UNITED STATES

## Abstract

Peroxisome proliferator-activated receptor γ (PPARγ) is a ligand-dependent transcription factor involved in many aspects of metabolism, immune response, and development. Total-body deletion of the two *Pparg* alleles provoked generalized lipoatrophy along with severe type 2 diabetes. Herein, we explore the appearance and development of structural and functional alterations of the kidney, comparing *Pparg* null-mice to their littermate controls (carrying *Pparg* floxed alleles). We show that renal hypertrophy and functional alterations with increased glucosuria and albuminuria are already present in 3 weeks-old *Pparg* null-mice. Renal insufficiency with decreased creatinine clearance progress at 7 weeks of age, with the advance of the type 2 diabetes. At 52 weeks of age, these alterations are accompanied by signs of fibrosis and mesangial expansion. More intriguingly, aged *Pparg* null-mice concomitantly present an anti-phospholipid syndrome (APS), characterized by the late appearance of microthrombi and a mesangioproliferative pattern of glomerular injury, associated with significant plasmatic levels of anti-β_2_- glycoprotein1 antibodies and renal deposition of IgG, IgM, and C3. Thus, in line with the role of PPARγ in metabolic homeostasis, *Pparg* null-mice first represent a potent model for studying the initiation and the development of diabetic nephropathy. Second, and in relation with the important PPARγ activity in inflammation and in immune system, these mice also highlight a new role for PPARγ signaling in the promotion of APS, a syndrome whose pathogenesis is poorly known and whose current treatment is limited to prevention of thrombosis events.

## Introduction

Diabetic nephropathy is one major complication of type 2 diabetes. In human, the injurious effects of hyperglycemia are separated into macrovascular complications (coronary artery disease, peripheral arterial disease, and stroke) and microvascular complications (diabetic nephropathy, neuropathy, and retinopathy). Diabetic nephropathy is currently the leading cause of end-stage renal disease in many countries and it occurs in ~30% of people with type 1 diabetes and 25–40% of people with type 2 diabetes. Its progression has been described in 5 steps, from an initial renal hypertrophy and hyperfiltration phase, which then persist with the occurrence of hyperglycaemia, followed by the appearance of microalbuminuria, the installation of progressive renal failure and finally an end-stage renal failure. Lack of satisfactory animal model has brought up the establishment of a list of criteria that should be met for tagging a kidney pathology with either a progressive diabetic nephropathy or an advanced states of diabetic nephropathy (Animal Models of Diabetic Complications Consortium (AMDCC) (http://www.amdcc.org).

Peroxisome proliferator-activated receptor γ (PPARγ) is a ligand-dependent transcription factor of the nuclear receptor superfamily, which plays a central role in adipogenesis and is expressed in different compartments of the kidney at both the glomerular and tubular levels [1]. In various rodent models of type 2 diabetes (db/db mice, obese Zucker rats, and OLETF rats), treatment with thiazolidinedione (TZD)–a high-affinity synthetic ligand for PPARγ –not only improves insulin resistance and glycemic control, but also ameliorates diabetic nephropathy by inhibiting glomerular hypertrophy, reducing mesangial matrix expansion, and improving proteinuria and renal function [2]. On the other hand, TZD provokes substantial renal sodium retention associated with edema and plasma volume expansion, the mechanisms of which remain unclear (reviewed in Horita et *al*. [3]). In addition, a distinct direct action of PPARγ in the kidneys was suggested by cell-specific deletion of *Pparg* in mouse macrophages, which triggers the appearance of lupus nephritis signs [4].

While cell-specific *Pparg* deletion is useful for identifying its numerous cell- and tissue-specific activities, the overall systemic role of *Pparg* can be better appreciated upon total deletion. Using an epiblast-specific Cre-mediated recombination of *Pparg* floxed alleles (*Pparg*^*fl*^*)*, we generated total-body *Pparg*-null mice [5], hereafter called *Pparg*^*Δ/Δ*^. These mice showed total absence of adipose tissue, early development of severe type 2 diabetes, and remarkable metabolic inflexibility that we explore in a separate report (Gilardi et al., manuscript in preparation).

In the present report, the early development of these severe metabolic alterations lead us to examine *Pparg*^*Δ/Δ*^ for the presence of microvascular complications that are highly prevalent in type 2 diabetes [6]. Altough the retina did not exhibit modifications that could be linked to type 2 diabetes, a systematic analysis of the kidney at different time points along development and aging identified the first marks of glomerular and tubular functional alterations as early as seven weeks of age, parallel to the development of the severe type 2 diabetes. Importantly, we demonstrate that aging mice developed an anti-phospholipid syndrome.

## Materials and methods

### Animals and clinical parameters

Animal care and treatments were performed in agreement with the guidelines established by the European Community Council Directives (86/609/EEC) and were authorized by the commission for animal experimentation of the cantonal veterinary services (Canton of Vaud). When needed, mice were sacrificed using CO2 inhalation. Genotype denomination follows as far as possible, the rules recommended by the Mouse Genome Database (MGD) Nomenclature Committee. Construction of the PPARγ floxed allele (initially called PPARγL2, hereafter called *Pparg*^*fl*^, and the *Pparg*-null allele resulting from Cre recombination (PPARγL-), hereafter called *Pparg*^***Δ***^) has previously been described [7]. The PPARγ null mice obtained via *Pparg* epiblast-specific deletion have been previously reported [5]. Briefly, since homozygous PPARγ-/- mice show an embryonic lethal phenotype due to a placental defect [5, 8], we crossed SOX2CRE^tg/+^ transgenic mouse strain with mice homozygous for the *Pparg* floxed allele (*Pparg*^fl/fl^) to generate epiblast-specific deletion, generating *Pparg*^fl^ deletion in all cells participating to the embryo development whilst preserving PPARγ expression in the trophoblast [5]. Normal placenta development allows SOX2CRE^tg/+^*Pparg*^*emΔ/Δ*^ (hereafter called *Pparg*^*Δ/Δ*^) pups to be born. *Pparg* expression is totally undetectable in *Pparg*^*Δ/Δ*^ embryos as well as in various adult tissues (including liver, kidney, and bones; data not shown). The control littermates (CTL) have no SOX2CRE transgene and two functional *Pparg* alleles (*Pparg*^*fl/+*^). Both male and female have been used throughout the study, except for the 52 week-old time point, for which only female were available due to a relative earlier death of male mice.

Functional parameters were evaluated at the Mouse Metabolic Evaluation Facility (MEF) of the University of Lausanne. Urinary measurements were done using spot urines or 24h urine collection from metabolic cages. Urinary albumin and glucose were evaluated with Albuwell M ELISA kit (Exocell, USA) and QuantiChrom^TM^ Glucose Assay Kit (BioAssay System, USA), respectively. At sacrifice, kidneys were rapidly snap-frozen or put in PFA 4% for the subsequent embedding in paraffin.

### Detection of autoantibodies

Plasma levels of anti-nuclear antibodies (ANAs) and anti-dsDNA IgGs were measured by commercially available ELISAs (Alpha Diagnostic International, USA). Antibodies anti β_2_-glycoprotein1 were determined by ELISA, coating a 96 well-plate with 1μg/well of recombinant mouse β_2_-glycoprotein1 (6575-AH-050, R&D Systems) overnight at 4°C. After blocking, mouse plasma samples (dil 1:100) were loaded and incubated at 37°C for 1h. The detection was done with HRP-conjugated anti mouse IgG-Fc antibody and TMB as substrate (Bethyl Laboratoires, USA).

### Angiography for retinal vessels

*Pparg*^*Δ/Δ*^ mice often showed corneal problems such as opacity due to the lack of Meibomian glands (data not shown). Thus, to attempt to maintain a transparency of the cornea, the animals were treated with Vismed for 7 days before investigating the eye fundus. Before each fluorescein angiography (FA), 28–32 week old mice were anesthetized by intraperitoneal injection of a solution of ketamine/xylazine (66.7 mg/kg / 9.88 mg/kg). Upon full dilation after instillation of Tropicamide (0.5%, SDU Faure/Théa) and Phenylephrine hypochloride (2.5%, Bausch Lomb Minims), animals were placed onto the positioning table. Then an ophthalmic gel (Goniovisc 2.5%, HUB Pharmaceuticals) was applied on the eye and the objective was oriented for imaging. Just after intraperitoneal injection of a solution of fluorescein (10 μL of 1% fluorescein in saline per gram of body weight), videos of the FA were recorded using a retinal imaging system allowing a high imaging resolution through a specific objective for mice (Micron III, Phoenix Research Labs, USA). Images were then extracted at early (3 min) and late (> 6 min) time-points and analyzed.

### Histology and morphometry

For histological analysis, paraffin sections were stained with haematoxylin and eosin (HE), periodic acid-Schiff (PAS), Silver methenamine, and Masson’s trichrome, according to routine protocols. Glomerular cross sectional area was assessed by tracing along the outline of the glomerular tuft of 60–90 glomeruli per mouse. For specific immunohistochemical staining for collagen IV deposition and macrophage infiltration, paraffin kidney sections were incubated overnight at 4°C with the following primary antibodies: rabbit anti collagen IV (dil 1:400, 2150–1470, AbD Serotec, Germany) and rat anti F4/80 (dil 1:800, ab6640, Abcam, USA). HRP-conjugated secondary antibodies were then applied, followed by DAB (Vector Laboratories, USA) as the chromogen. After counterstaining with haematoxylin, all the sections were examined by light microscopy (Microscope Zeiss Imager A1, Carl Zeiss Ltd., United Kingdom) and images were collected at 40x magnification. Collagen IV, expressed as a percentage of stained area, and F4/80 quantification, expressed as number of positive cells/glomerular cross-section, were then evaluated in 40–60 glomeruli from each section. Expression of collagen IV in tubulointerstitium was instead measured in 20x images in the entire cortical area.

IgG, IgM and C3 were stained by Fc-specific anti mouse IgG, μ-chain specific anti mouse IgM (FITC conjugated, Sigma, USA), and anti C3 antibody (ab11862, Abcam), respectively.

### Real-time quantitative PCR

Total RNA was isolated after homogenisation in TRI-Reagent (Ambion, Thermo Fisher Scientific Inc.) and extraction with RNeasy Mini Kit (Qiagen). Gene expression was analysed by real-time quantitative PCR (FastStart Universal SYBR Green Master, Roche, USA) in a Stratagene MX3005P Detection System (Agilent Technologies, USA). Ribosomal protein S9 (*RPS9*) was used as housekeeping gene. Primer sequences are available upon request.

### Statistical analysis

Values, expressed as mean ± SEM, were analysed using Prism 5.0 (GraphPad Software, USA). Student’s *t* test, or two-way ANOVA with Bonferroni post-test for multiple group comparisons were used to assess statistical significance. A P value <0.05 was considered statistically significant.

## Results

### Lack of retinal damages in *Pparg*^*Δ/Δ*^ mice

Diabetic retinopathy is a microvascular complication of diabetes. It is characterized by various retinal lesions and represents the leading cause of blindness in the working population [6]. Considering the severity of type-2 diabetes in *Pparg*^*Δ/Δ*^ mice, we explored the possible development of diabetes associated retinal diseases, using fluorescein injection in the periphery. As compared to control mice, no morphological abnormalities or leakage of the dye were observed in mutant mice at 28 weeks suggesting the presence of an intact inner blood-retinal barrier (S1 Fig). Similar observations were obtained in 46 weeks old mice (data not shown) indicating that *Pparg*^*Δ/Δ*^ mice do not develop diabetic retinopathy.

### *Pparg*^*Δ/Δ*^ mice present early signs of glomerular nephropathy

The appearance of renal alterations in *Pparg*^*Δ/Δ*^ mice was in contrast precocious and obvious. At the time of weaning (3 weeks), *Pparg*^*Δ/Δ*^ mice exhibited metabolic disorders with elevated glycemia in the fed state (Table 1). At the same age, plasma creatinine was unchanged, but *Pparg*^*Δ/Δ*^ mice presented renal hypertrophy, increased glucosuria, and 5.6-fold higher albuminuria compared to control mice, suggesting that kidney functionality was already altered. At 7 weeks of age, evaluation of young mice in metabolic cages revealed significantly increased water intake and urinary output compared to control mice (Table 1). At this time-point, severely impaired *Pparg*^*Δ/Δ*^ renal function was based on increased plasma creatinine and urea nitrogen, and reduced creatinine clearance (40% that of littermate controls). Moreover, 24-hour urinary collection revealed massive glucosuria and 16-fold higher albuminuria in *Pparg*^*Δ/Δ*^ mice compared to controls (Table 1). The marked glucosuria led to osmotic diuresis, largely explaining the high urinary output, while the concomitant albuminuria reflected glomerular damages. Finally, *Pparg*^*Δ/Δ*^ mice showed greater sodium and potassium elimination compared to control mice. This may be partly due to the hyperphagia of the lipodystrophic mice, which showed very low circulating leptin. However, the difference remained important after correction per gram of food intake, pointing to a decrease in tubular reabsorption (S2 Fig).

**Table 1:** Functional renal parameters of control and *Pparg*^*Δ/Δ*^ mice at 3 and 7 weeks of age.

Parameters	CTL	*Pparg*^*Δ/Δ*^	P value
**3 weeks**			
Body weight (g)	12.36 ± 0.39(N = 7)	8.30 ± 0.44(N = 5)	<0.0001
Glycemia (mmol/L)	10.05 ± 0.25(N = 11)	15.97 ± 2.20(N = 10)	0.0113
Total kidney w./body w. (x100)	1.24 ± 0.03(N = 7)	1.69 ± 0.06(N = 5)	<0.0001
Plasma creatinine (μmol/L)	10.4 ± 0.24(N = 10)	11.2 ± 0.9(N = 8)	0.4088
Glucosuria/creatininuria (mg/mg)	2.76 ± 0.39(N = 6)	7.16 ± 1.91(N = 4)	0.0241
Urine albumin/creatinine (μg/mg)	53.35 ± 7.77(N = 6)	296.50 ± 87.31(N = 4)	0.0082
**7 weeks**			
Body weight (g)	23.68 ± 0.28(N = 7)	23.02 ± 0.82(N = 6)	0.4383
24h water intake (ml)	5.17 ± 0.43(N = 7)	30.51 ± 0.78(N = 7)	<0.0001
24h urinary output (ml)	1.05 ± 0.10(N = 7)	26.33 ± 1.3(N = 6)	<0.0001
Plasma Urea Nitrogen (mmol/L)	8.0 ± 1.10(N = 5)	11.10 ± 1.10(N = 4)	0.0043
Plasma creatinine (μmol/L)	8.77 ± 0.57(N = 7)	19.61 ± 1.45(N = 7)	<0.0001
Creatinine clearance (ml/min)	0.498 ± 0.060(N = 7)	0.189 ± 0.025(N = 6)	0.0009
Urine glucose (mg/24h)	1.64 ± 0.15(N = 7)	2273.03 ± 144.30(N = 6)	<0.0001
Urine albumin (μg/24h)	62.86 ± 8.78(N = 4)	1025.28 ± 52.03(N = 4)	<0.0001

Functional parameters of control (CTL) and *Pparg*^*Δ/Δ*^ mice at 3 and 7 weeks of age. Values are expressed as means ± SEM; N = number of animals; w. = weight.

**Fig 1:**
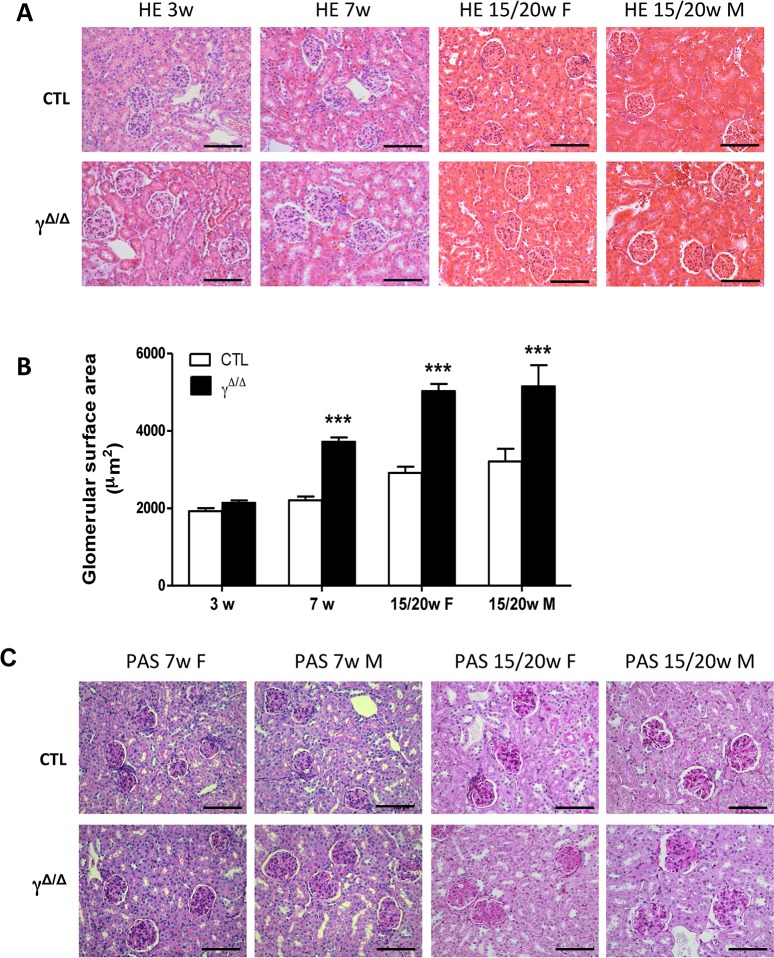
Early histomorphometric damages in *Pparg*^*Δ/Δ*^ kidney. (A) Representative images of paraffin kidney sections stained with HE in control (CTL) and *Pparg*^*Δ/Δ*^ mice (γ^*Δ/Δ*^) at different ages. Scale bar represents 100μm (B) Quantitative analysis of glomerular cross-sectional area in control and *Pparg*^*Δ/Δ*^ mice. 3 weeks: 5 controls and 5 *Pparg*^*Δ/Δ*^ P>0.05. 7 weeks: 6 controls and 4 *Pparg*^*Δ/Δ*^ P<0.0001. 15/20 weeks females: 4 controls and 4 *Pparg*^*Δ/Δ*^ P<0.0001. 15/20 weeks males: 3 controls and 3 *Pparg*^*Δ/Δ*^ P<0.0001 Data are expressed as mean ± SEM. ***P<0.001 *Pparg*^*Δ/Δ*^ *vs*. control littermates (C) Representative sections of kidney sections from male and female *Pparg*^*Δ/Δ*^ mice and littermate controls stained with periodic acid-Schiff (PAS). Scale bar represents 100μm.

We next performed systematic histopathology analyses of the kidneys of *Pparg*^*Δ/Δ*^ mice at different time-points. Quantitative analyses of kidney sections at 3, 7, and 15–20 weeks of age revealed an age-associated increase of the glomerular cross-sectional area in *Pparg*^*Δ/Δ*^ mice. At 7 weeks of age, this area was already significantly higher compared to control littermates (Fig 1A and 1B). At 15–20 weeks of age, males showed glomerular lesions with moderate-to-severe widening of the mesangial areas, together with moderate mesangial cell proliferation. Females showed similar glomerular lesions at this age, although the changes were generally less severe (Fig 1C). At this time-point, *Pparg*^*Δ/Δ*^ mice did not exhibit nodular glomerulosclerosis and there were no specific findings in the tubulointerstitial compartment.

Thus, within the context of severe type 2 diabetes, the renal functional alterations and histological modifications in *Pparg*^*Δ/Δ*^ mice were consistent with an early stage of diabetic nephropathy.

### Chronic kidney damages in *Pparg*^Δ/Δ^ ageing mice associate diabetic nephropathy and antiphospholipid syndrome

**Fig 2:**
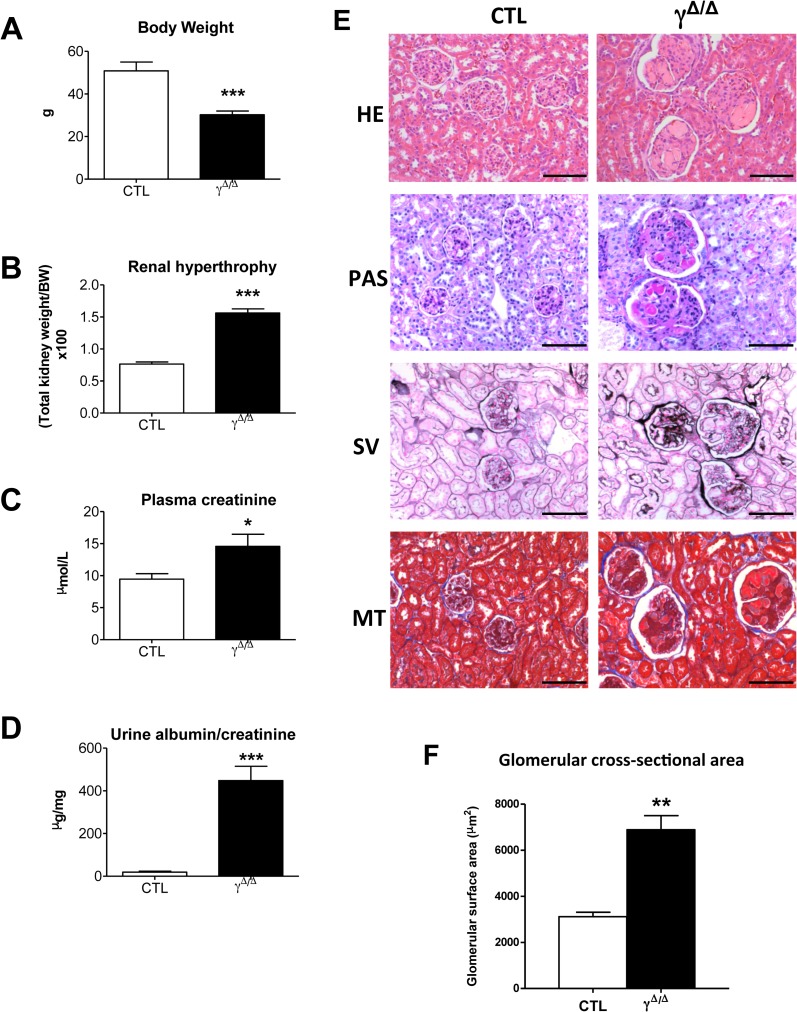
Chronic functional and histological damages in ageing *Pparg*^*Δ/Δ*^ kidney. Indicated parameters and histological studies were performed in 52 weeks old control (CTL) and *Pparg*^*Δ/Δ*^ (γ^*Δ/Δ*^) mice. (A) Body weight (6 controls and 9 *Pparg*^*Δ/Δ*^ P = 0.0002), (B) renal hypertrophy as evaluated by the ratio total kidney weight over total body weight (X100) (6 controls and 9 *Pparg*^*Δ/Δ*^ P<0.0001), (C) plasma creatinine (6 controls and 7 *Pparg*^*Δ/Δ*^ P = 0.0384), (D) albuminuria (7 controls and 9 *Pparg*^*Δ/Δ*^ P<0.0001) (E) Representative images of paraffin kidney sections stained with haematoxylin and eosin (HE), Periodic Acid Shiff (PAS), Silver methenamine (SV), and Masson’s trichrome (MT). Scale bar represents 100μm. (F) glomerular cross-sectional area in 52 weeks female mice (4 controls and 5 *Pparg*^*Δ/Δ*^ P = 0.0011). Data in the graphs corresponds to the mean ± SEM. *P<0.05, **P<0.01, ***P<0.001 *Pparg*^*Δ/Δ*^ *vs*. age matched control littermates.

Searching for specific signs of diabetic nephropathy, we evaluated the evolution of the lesions with increasing age. At 52 weeks, *Pparg*^*Δ/Δ*^ mice exhibited altered renal functions based on renal hypertrophy, plasma creatinine, and albuminuria (Fig 2A–2D) as well as extensive glomerular changes, including increased glomerular size (Fig 2E and 2F). Periodic acid-Schiff (PAS) staining revealed increased mesangial matrix deposition and mesangial hypercellularity and some glomeruli adopted a lobular shape. Moreover, both PAS and silver methenamine stainings suggested increased glomerular basement membrane thickness (Fig 2E).

**Fig 3:**
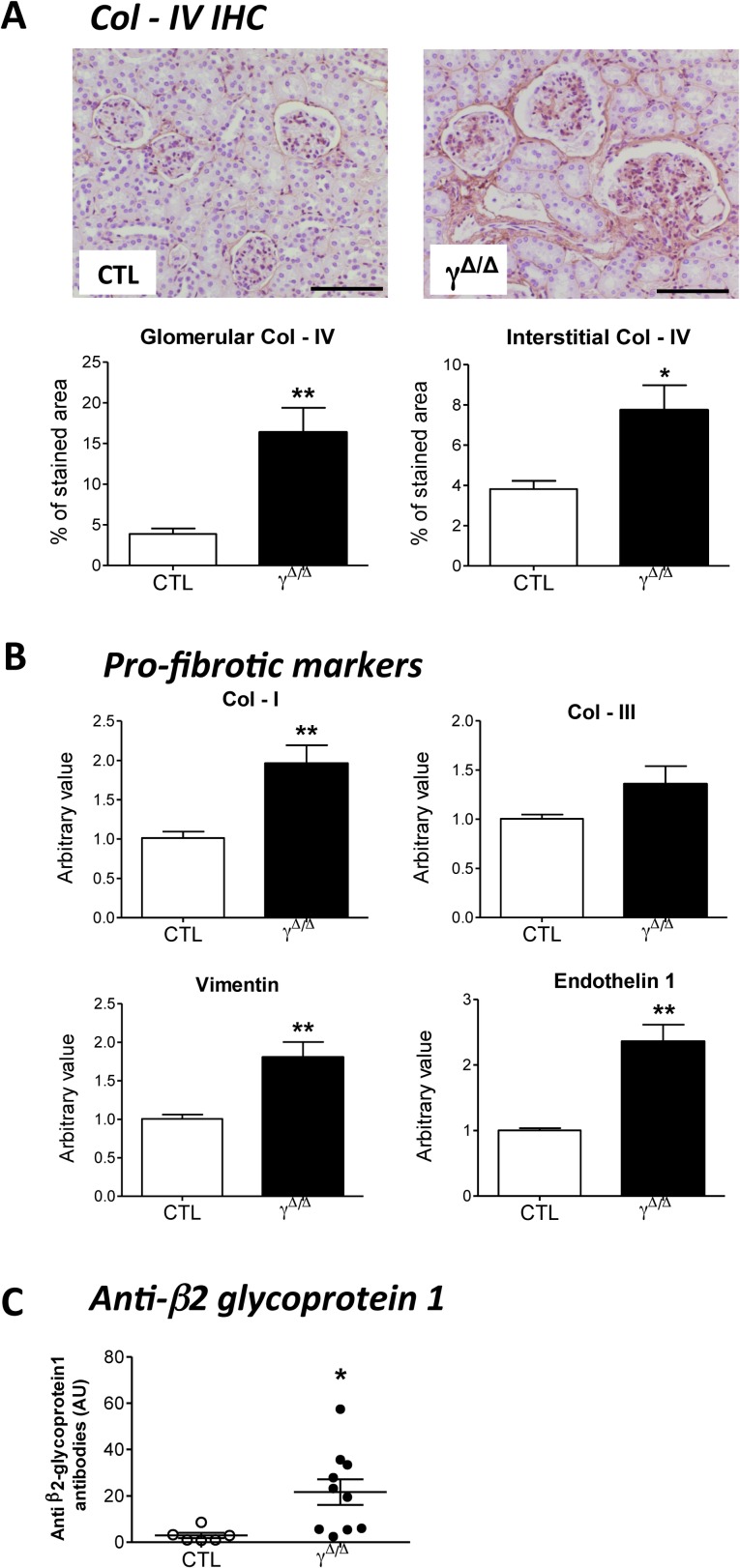
Immunohistochemistry and gene expression of fibrosis markers in kidney of ageing *Pparg*^*Δ/Δ*^ mice, and plasmatic levels of anti-β_2_-glycoprotein1 antibodies. 52 weeks old control (CTL) and *Pparg*^*Δ/Δ*^ (γ^*Δ/Δ*^) animals were analyzed for the following parameters: (A) *Top panels*: Kidney Immunohistochemistry for collagen IV (Col-IV). Positive staining is in brown. Scale bar represents 100μm. *Bottom panels*: quantification of Col-IV deposition at glomerular levels (4 controls and 4 *Pparg*^*Δ/Δ*^ P = 0.0067) and tubulointerstitial levels (4 controls and 4 *Pparg*^*Δ/Δ*^ P = 0.0238), (B) Evaluation by RT-qPCR of the following gene expression in the kidney of CTL (N = 5) and *Pparg*^*Δ/Δ*^ (N = 7) mice: collagen I (Col-I, P = 0.0081), collagen III (Col-III, P = 0.1441, not significant), vimentin (P = 0.0083) and Endothelin 1 (P = 0.0015). Results are reported as fold change with respect to control levels, which were arbitrarily set to 1. (C). Plasma levels of antibodies against the β_2_-glycoprotein1 (6 controls and 10 *Pparg*^*Δ/Δ*^ P = 0.0233). Data in the graphs corresponds to the mean ± SEM. *P<0.05, **P<0.01 *Pparg*^*Δ/Δ*^ *vs*. age matched control littermates.

Diabetic nephropathy is generally characterized by tubulointerstitial fibrosis, and accumulation of extracellular matrix components in the glomerular basement membrane and mesangium [9, 10]. Real-time qPCR—revealed moderate but significant increase in collagen I and III expression at 7 weeks (S3 Fig). In aged *Pparg*^*Δ/Δ*^ mice, immunohistochemistry for collagen IV (Fig 3A) and the increased renal expressions of collagen I, vimentin, and endothelin1 (Fig 3B) suggested that fibrosis continued throughout aging, consistent with the concomitant mesangial expansion.

Intriguingly the kidneys of aged *Pparg*^*Δ/Δ*^ mice showed the appearance of nodular lesions consisting of accumulated amorphous material within numerous glomeruli. The lesions were strongly PAS-positive, non-argyrophilic, negative for Congo red staining excluding amyloid deposition (data not shown), and a Masson’s trichrome staining pattern indicating presence of intracapillary thrombi (Fig 2E). The incidence and large size of the thrombi led us to suspect an antiphospholipid syndrome (APS)–i.e., an autoimmune disease diagnosed based on clinical presence of thrombotic events and persistent positivity for antiphospholipid antibodies, particularly anti-β_2_-glycoprotein1 [11, 12]. The kidney is a major target of APS, with potential thrombosis at any level [12, 13]. In line with APS criteria, 52-week-old *Pparg*^*Δ/Δ*^ mice showed renal thrombosis and significantly elevated plasma levels of anti-β_2_-glycoprotein 1 antibodies (Fig 3C).

**Fig 4:**
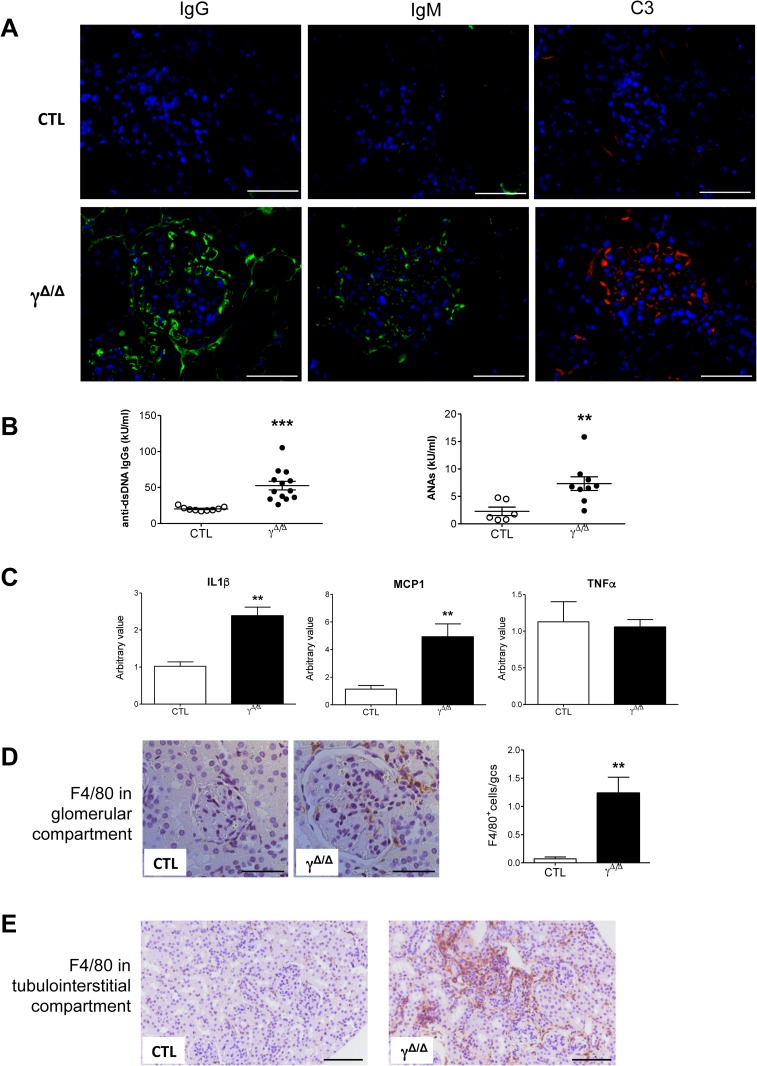
Renal immune complex deposition and inflammation in old *Pparg*^*Δ/Δ*^ mice. (A) Immunofluorescence for IgG (left panel, green staining), IgM (middle panel, green staining), and C3 (right panel, red staining) deposition on frozen kidney sections of 52 weeks old control (CTL) and *Pparg*^*Δ/Δ*^ (γ^*Δ/Δ*^) mice. The nuclei are labeled in blue (DAPI). Scale bar represents 50μm. (B) Plasma levels of anti dsDNA IgGs (9 controls and 13 *Pparg*^*Δ/Δ*^ P = 0.0003) and ANAs (6 controls and 9 *Pparg*^*Δ/Δ*^ P = 0.0098). (C) Evaluation of IL1β (5 controls and 7 *Pparg*^*Δ/Δ*^ P = 0.001), MCP1 (5 controls and 7 *Pparg*^*Δ/Δ*^ P = 0.0079), and TNFα (5 controls and 6 *Pparg*^*Δ/Δ*^ P = 0.799, not significant) gene expression by RT-qPCR in 52 weeks old control and *Pparg*^*Δ/Δ*^ animals. Results are reported as fold change with respect to control levels arbitrarily set to 1. Data are the mean ± SEM (D) The two left panels are representative images of F4/80 immunohistochemistry in glomeruli (scale bar represents 50μm). The right panel is a relative quantification of positive cells (stained in brown; 5 controls and 7 *Pparg*^*Δ/Δ*^ P = 0.0062). (E) Representative images of F4/80 immunohistochemistry in tubulointerstitial areas (scale bar represents 100μm) of 52 weeks old control and *Pparg*^*Δ/Δ*^ mice. *P<0.05, **P<0.01 *Pparg*^*Δ/Δ*^ *vs*. control littermates.

APS is classified as primary when it occurs alone, and as secondary when it occurs in association with other autoimmune disorders, most frequently systemic lupus erythematosus (SLE). Considering that cell-specific *Pparg* deletion in macrophages causes the appearance of SLE signs [4], we further extended our investigations to search for renal signs of SLE. No changes in IgG or IgM were observed at 25 weeks of age (data not shown). However, 52-week-old *Pparg*^*Δ/Δ*^ mice showed strong glomerular deposition of IgG, IgM, and C3 (Fig 4A). Consistently, 52-week-old *Pparg*^*Δ/Δ*^ mice showed increased circulating anti-dsDNA IgGs and antinuclear antibodies (ANAs) compared to control littermates (Fig 4B), further confirming the autoimmune disorder [14]. Finally, the associated inflammation [15] was indicated by a statistically significant upregulation of IL1β and MCP1 in the kidneys of 52-week-old *Pparg*^*Δ/Δ*^ mice, whereas TNFα remained unchanged (Fig 4C). Accordingly, increased macrophage infiltration was detected in both the glomerular (Fig 4D) and tubulointerstitial compartments (Fig 4E).

## Discussion

In summary, this report highlights two major kidney features of *Pparg*^*Δ/Δ*^ mice. The first observation in this model of total lipodystrophy was that early signs of type 2 diabetes led to early alterations of kidney function, which progressively and rapidly worsened into renal insufficiency with massive albuminuria. An association between lipodystrophy and diabetic nephropathy has been previously reported in lipoatrophic A-ZIP/F-1 mice [16, 17] and in *ob/ob* mice carrying deletion of *Pparg2* (a *Pparg* splice variant mainly expressed in adipose tissue) [18]. The present lack of a satisfactory animal model for diabetic nephropathy has led the research community to propose a series of criteria that a model should meet to be useful in translational research (Animal Models of Diabetic Complications Consortium; AMDCC) [10]. While *Pparg*^*Δ/Δ*^ mice fulfil the criteria of functional alterations, they lack some histopathological features required to fully mimic human diabetic nephropathy. However, we believe that this model is particularly pertinent for mechanistic studies of the initial perturbations in diabetic nephropathy.

The second feature noted was the appearance in aged mice of APS in association with signs of lupus nephritis. To our knowledge, the *Pparg*^*Δ/Δ*^ mouse is the first mouse model that spontaneously develops APS, representing a new tool for exploring the unsolved questions related to APS pathogenesis. The importance of PPARγ in immunity and inflammation is well proven [19, 20] and both aspects may be of importance for APS development.

The link between APS and autoimmune diseases is well established. Anti-phospholipid antibodies are indeed widely considered as the main actors in inducing the pathological coagulation process, leading to thrombosis, the devastating clinical issue in APS that can potentially affect any organ in the body. In humans, while the causes of primary APS are poorly known [13], the main cause of secondary APS is SLE, affecting around 30% of APS patients [21]. Kidney alterations appear equally in primary and secondary APS. In both forms, patient treatment is mainly limited to preventing thrombosis events. Consistent with the role of PPARγ in inflammation and immunity, TZD treatment is known to be protective against complications such as atherosclerosis and renal disease in various murine SLE models [22–24]. Our results raise the question whether TZD treatment would also help in controlling APS complication in kidney.

The link between APS and inflammation is in contrast more controversial. Some classifications consider the lack of inflammatory context as one criteria for APS diagnostic [25], whereas recent reports insist on the pro-inflammatory status of APS [26, 27]. Recently, NF-kB and c-Jun/AP-1 have been involved in the pathogenic role of β_2_-glycoprotein1- antibodies as important mediators in the production of prothrombotic and proinflammatory molecules in endothelial cells and monocytes [28]. Along this line, specific deletion of PPARγ in macrophages led to a dramatic increase in basal levels of proinflammatory molecules, possibly through NF-kB-mediated signalling [29]. According to our findings, and albeit one limitation of our study is the lack of human studies, we hypothesise that PPARγ whole body deletion affects macrophage functions, resulting in a deficient clearance of injured cells, a deleterious alteration of the normal physiological levels and function of the β_2_-glycoprotein1- antibody complexes, thereby promoting a prothrombotic status. Such a mechanism would possibly contribute to primary APS, considering that while heterogeneous antiphospholipid antibodies can be transiently detected after infections in the general population, the mechanisms leading to their permanent circulation are still unknown [13].

One particular feature of our model, and one of its limitation, is the association of a severe type 2 diabetes and sign of lupus nephritis. In humans, although the overlap between SLE and type-2 diabetes is uncommon (0.8%) [30], emerging evidences point to an increased predisposition of developing insulin resistance and metabolic syndrome in SLE patients and in mouse models [31, 32]. This combination of diseases and the double jeopardy of subsequent complications underscore the need to identify novel and safe treatments. Finally, considering that only two cases of human partial lipodystrophy associated with antiphospholipid and anticardiolipin antibodies were reported [33, 34], it is likely that specific PPARγ deletion at cellular level, rather than the lipoatrophy, is fundamental in favoring the autoimmune response.

In conclusion, the dual kidney phenotype observed herein reflects two major systemic activities of PPARγ, one relating to metabolic homeostasis (specifically, glucose and lipid metabolism) and the other to immunity and inflammation. The intricate renal consequences of the total lack of *Pparg*^*Δ/Δ*^ undescore the need to comprehensively understand PPARγ systemic actions when this nuclear receptor is targeted. In clinical practice TZDs are known to ameliorate many aspects of diabetic as well as non-diabetic nephropathy [1]. Further research should focus on the immunological aspect, particularly with regards to the present poor palette of therapeutic approaches for APS.

## Supporting information

S1 Fig Evaluation of vascular integrity in the retina of *Pparg^Δ/Δ^* mice. Eye fundus of (A, B) control (CTL) and (C, D) *Pparg*^*Δ/Δ*^ (γ^*Δ/Δ*^) mice. No distinct morphological alterations are observed between the two groups. Fluorescein angiography (FA) shows a normal vascular pattern and no leakage of the vessels in (C’, D’) *Pparg*^*Δ/Δ*^ mice compared to (A’, B’) control littermates. Nonetheless, a certain diffusion of the signal occurs in the KO mice due to some opacity of the cornea.
(TIF)

S2 Fig Evaluation of sodium and potassium clearance and fractional excretion in 7 weeks old *Pparg^Δ/Δ^* mice. Values are expressed as means ± SEM; Natriuria: 7 controls and 6 *Pparg*^*Δ/Δ*^ P = 0.0028; Plasma Sodium: 7 controls and 7 *Pparg*^*Δ/Δ*^ P = 0.0216; Clearance Sodium: 7 controls and 6 *Pparg*^*Δ/Δ*^ P<0.0001; FE (Sodium): 7 controls and 6 *Pparg*^*Δ/Δ*^ P = 0.0001; Kaliuria: 7 controls and 6 *Pparg*^*Δ/Δ*^ P = 0.0418; Plasma Potassium: 7 controls and 7 *Pparg*^*Δ/Δ*^ P = 0.013; Clearance Potassium: 7 controls and 6 *Pparg*^*Δ/Δ*^ P = 0.0013; FE (Potassium): 7 controls and 6 *Pparg*^*Δ/Δ*^ P = 0.0001. FE: fractional excretion. *P<0.05, **P<0.01, ***P<0.001 *Pparg*^*Δ/Δ*^ *vs*. control littermates
(TIF)

S3 Fig Fibrosis and inflammation parameters in 7 weeks old *Pparg^Δ/Δ^* mice. (A) RT-qPCR in 7 weeks old control (CTL, N = 5) and *Pparg*^*Δ/Δ*^ (γ^*Δ/Δ*^; N = 5) animals to evaluate gene expression of collagen I (Col–I, P = 0.0062), collagen III (Col–III; P = 0.0165) and vimentin (P = 0.0299). Results are reported as fold change with respect to control levels, which were arbitrarily set to 1. Data show mean ± SEM. (B) Evaluation by RT-qPCR in 7 weeks old control (N = 4) and *Pparg*^*Δ/Δ*^ (N = 5) animals of gene expression of IL1β (P = 0.1885; not significant) and MCP1 (P = 0.0312). Results are reported as in A. *P<0.05 and **P<0.01 *Pparg*^*Δ/Δ*^ *vs*. control littermates.
(TIF)
