# Supplementary figures and images for: Nephropathy in Pparg-null mice highlights PPARγ systemic activities in metabolism and in the immune system
Source: PLoS One. 2017 Feb 9;12(2):e0171474. doi: 10.1371/journal.pone.0171474 (PMC5300244; doi:10.1371/journal.pone.0171474)

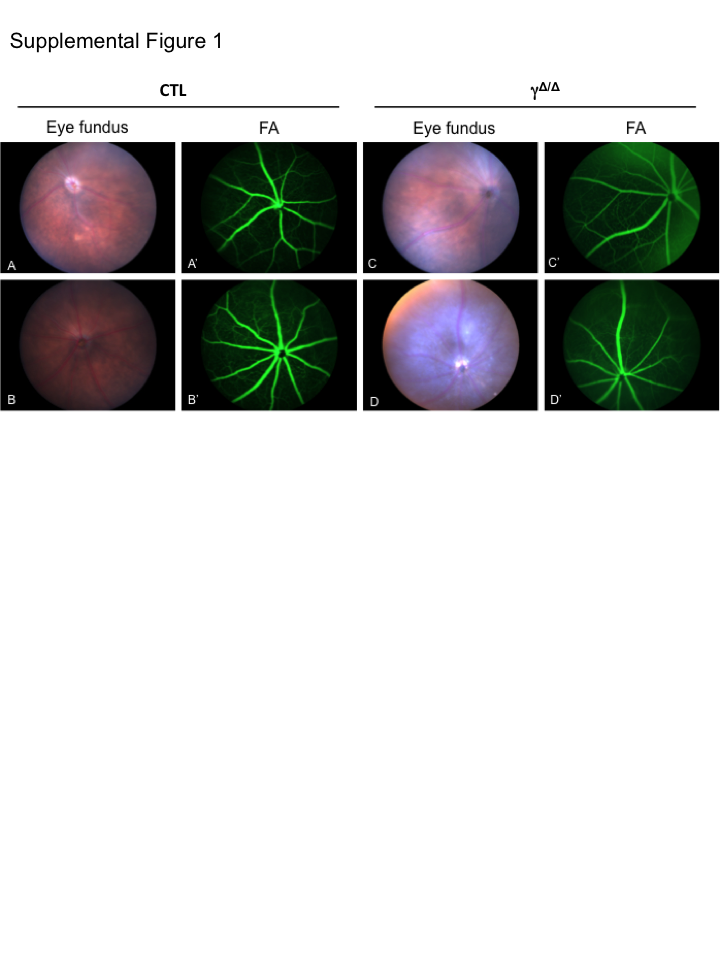

Supplement: S1 Fig — Eye fundus of (A, B) control (CTL) and (C, D) PpargΔ/Δ (γΔ/Δ) mice. No distinct morphological alterations are observed between the two groups. Fluorescein angiography (FA) shows a normal vascular pattern and no leakage of the vessels in (C’, D’) PpargΔ/Δ mice compared to (A’, B’) control littermates. Nonetheless, a certain diffusion of the signal occurs in the KO mice due to some opacity of the cornea. (TIF) [file pone.0171474.s001.tif]

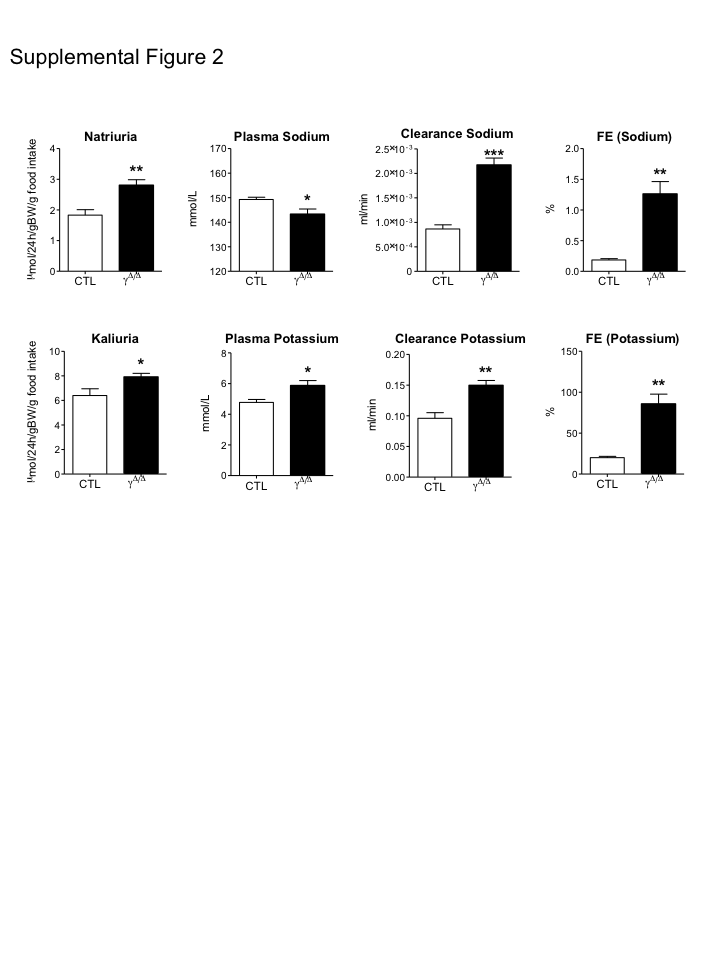

Supplement: S2 Fig — Values are expressed as means ± SEM; Natriuria: 7 controls and 6 PpargΔ/Δ P = 0.0028; Plasma Sodium: 7 controls and 7 PpargΔ/Δ P = 0.0216; Clearance Sodium: 7 controls and 6 PpargΔ/Δ P<0.0001; FE (Sodium): 7 controls and 6 PpargΔ/Δ P = 0.0001; Kaliuria: 7 controls and 6 PpargΔ/Δ P = 0.0418; Plasma Potassium: 7 controls and 7 PpargΔ/Δ P = 0.013; Clearance Potassium: 7 controls and 6 PpargΔ/Δ P = 0.0013; FE (Potassium): 7 controls and 6 PpargΔ/Δ P = 0.0001. FE: fractional excretion. *P<0.05, **P<0.01, ***P<0.001 PpargΔ/Δ vs. control littermates (TIF) [file pone.0171474.s002.tif]

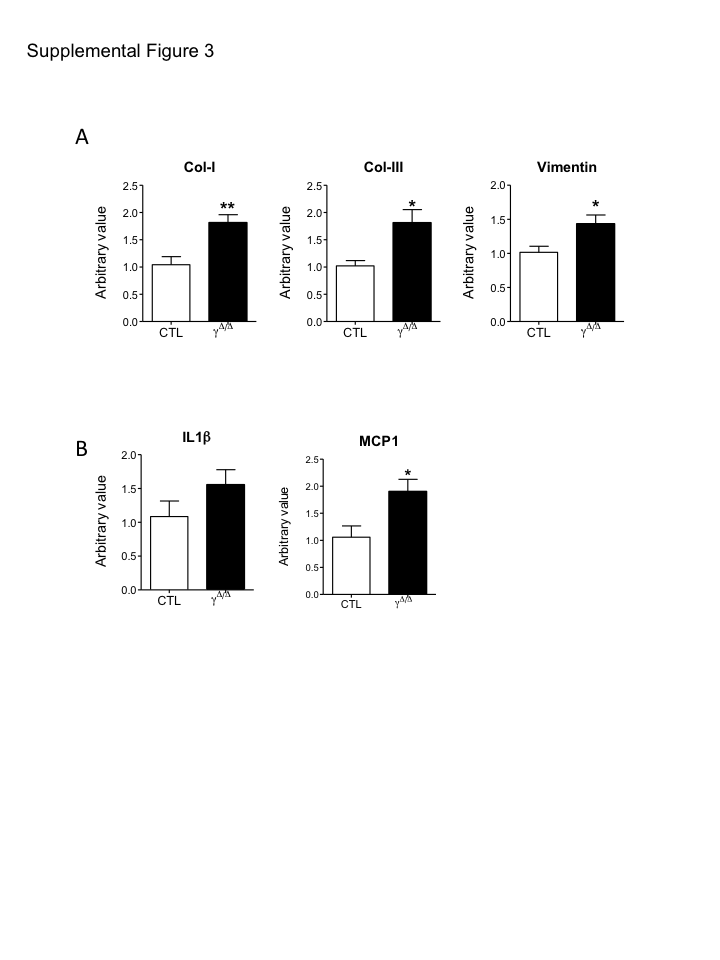

Supplement: S3 Fig — (A) RT-qPCR in 7 weeks old control (CTL, N = 5) and PpargΔ/Δ (γΔ/Δ; N = 5) animals to evaluate gene expression of collagen I (Col–I, P = 0.0062), collagen III (Col–III; P = 0.0165) and vimentin (P = 0.0299). Results are reported as fold change with respect to control levels, which were arbitrarily set to 1. Data show mean ± SEM. (B) Evaluation by RT-qPCR in 7 weeks old control (N = 4) and PpargΔ/Δ (N = 5) animals of gene expression of IL1β (P = 0.1885; not significant) and MCP1 (P = 0.0312). Results are reported as in A. *P<0.05 and **P<0.01 PpargΔ/Δ vs. control littermates. (TIF) [file pone.0171474.s003.tif]
